# PROLONGED NIGHTLY FASTING AMONG OLDER ADULTS: A PILOT STUDY EXPLORING CHANGES IN COGNITIVE FUNCTION

**DOI:** 10.1093/geroni/igac059.2960

**Published:** 2022-12-20

**Authors:** Dara James, Dorothy Sears, Linda Larkey, Molly Maxfield, Edward Ofori, Seung Yong Han, Kate Alperin, Peyton Osha

**Affiliations:** University of South Alabama, Mobile, Alabama, United States; Arizona State University, Phoenix, Arizona, United States; Arizona State University, Phoenix, Arizona, United States; Arizona State University, Phoenix, Arizona, United States; Arizona State University, Phoenix, Arizona, United States; Arizona State University, Phoenix, Arizona, United States; Arizona State University, Phoenix, Arizona, United States; Arizona State University, Phoenix, Arizona, United States

## Abstract

Aging is significantly associated with cognitive decline. A growing number of US adults ages ≥ 65 years have neurocognitive impairment resulting in compromised immediate and/or long-term health outcomes. Interventions to mitigate cognitive decline and promote healthy aging are needed. Research in intermittent fasting (IF) suggests positive health outcomes related to improvements in circadian rhythm and metabolism, which influence cognition. IF regimens may therefore result in improved neurocognitive health. We conducted an IF single-group, pre/post pilot study to explore changes in neurocognitive. Older adults (≥65 years of age; Nf18) with self-reported memory decline engaged in an 8-week, remotely-delivered, prolonged nightly fasting (PNF) intervention (14-hour nightly fasting, 10-hour daytime eating window). Our primary exploratory outcome was 8-week change in neurocognitive function assessed via composite score of the Memory and Attention Phone Screener (MAPS). Trends in outcome change were assessed with paired t-tests. Participants were mean age 69.7 years, non-Hispanic White, predominantly female (94%), married (50%), and employed (65%). Completion defined as percentage of participants that completed the intervention from those that started the intervention; completion rate was 90%. Paired t-test indicated a significant increase in scores on a neurocognitive screen (MAPS) pre/post-intervention (p=0.02) with a medium effect size (Cohen’s d=0.58). Findings suggest that PNF, a type of IF regimen targeted to align food intake with circadian rhythms, may significantly improve neurocognitive function among older adults with self-reported memory decline. These promising pilot results suggest a need for fully-powered, randomized controlled trials to test the efficacy of this non-pharmacological, low cost-to-burden ratio intervention.

